# Effects of surface functionalization of hydrophilic NaYF_4_ nanocrystals doped with Eu^3+^ on glutamate and GABA transport in brain synaptosomes

**DOI:** 10.1007/s11051-017-3958-8

**Published:** 2017-08-04

**Authors:** Bartlomiej Sojka, Daria Kociołek, Mateusz Banski, Tatiana Borisova, Natalia Pozdnyakova, Artem Pastukhov, Arsenii Borysov, Marina Dudarenko, Artur Podhorodecki

**Affiliations:** 1 Department of Experimental Physics, Wroclaw University of Science and Technology, Wyb. Wyspianskiego 27, 50-370 Wroclaw, Poland; 2 Department of Neurochemistry, Palladin Institute of Biochemistry, NAS of Ukraine, 9 Leontovicha str, Kiev, 01601 Ukraine

**Keywords:** Lanthanide doped fluoride nanocrystals, Glutamate, GABA, Na^+^-dependent uptake, Membrane potential, Brain nerve terminals, Neuroscience

## Abstract

Specific rare earth doped nanocrystals (NCs), a recent class of nanoparticles with fluorescent features, have great bioanalytical potential. Neuroactive properties of NaYF_4_ nanocrystals doped with Eu^3+^ were assessed based on the analysis of their effects on glutamate- and γ-aminobutyric acid (GABA) transport process in nerve terminals isolated from rat brain (synaptosomes). Two types of hydrophilic NCs were examined in this work: (i) coated by polyethylene glycol (PEG) and (ii) with OH groups at the surface. It was found that NaYF_4_:Eu^3+^-PEG and NaYF_4_:Eu^3+^-OH within the concentration range of 0.5–3.5 and 0.5–1.5 mg/ml, respectively, did not influence Na^+^-dependent transporter-dependent l-[^14^C]glutamate and [^3^H]GABA uptake and the ambient level of the neurotransmitters in the synaptosomes. An increase in NaYF_4_:Eu^3+^-PEG and NaYF_4_:Eu^3+^-OH concentrations up to 7.5 and 3.5 mg/ml, respectively, led to the (1) attenuation of the initial velocity of uptake of l-[^14^C]glutamate and [^3^H]GABA and (2) elevation of ambient neurotransmitters in the suspension of nerve terminals. In the mentioned concentrations, nanocrystals did not influence acidification of synaptic vesicles that was shown with pH-sensitive fluorescent dye acridine orange, however, decreased the potential of the plasma membrane of synaptosomes. In comparison with other nanoparticles studied with similar methodological approach, NCs start to exhibit their effects on neurotransmitter transport at concentrations several times higher than those shown for carbon dots, detonation nanodiamonds and an iron storage protein ferritin, whose activity can be registered at 0.08, 0.5 and 0.08 mg/ml, respectively. Therefore, NCs can be considered lesser neurotoxic as compared to above nanoparticles.

## Introduction

Nowadays, many efforts in research and development are focused on the design of nanomaterials with multiple functions, particularly those that can be used in theranostics, a new branch of nanomedicine that combines diagnostic and disease treatment modalities. Properties of nanocrystals (NCs) and nanoparticles often differ from those in bulk forms, thereby providing unexpected physical and chemical properties, and so a detailed understanding of principles of nanoparticle interaction with the cells is of value.

Rare earth ion doped NCs, a recently discovered class of nanoparticles with fluorescent properties, are promising structures to overcome problems of traditional fluorophores such as organic dyes and fluorescent proteins, which suffer from rapid photobleaching, spectral cross talking, blinking and small Stokes shift. Moreover, they have low immunotoxicity (Sojka et al. 2014). Recently, the authors of this study have obtained sub-10-nm fluoride NCs doped with Eu^3+^ ions, which are characterized by several emission bands, including a band at 720 nm depending on the Eu^3+^ concentration (Podhorodecki et al. 2013). Body tissues are permeable to infrared radiation outside the water absorbance region, and so the deep red imaging in vivo technologies are of great significance for medicine (Sojka et al. 2016). Rare earth ion doped NCs demonstrated unique optical properties and a potential to serve as excellent near-infrared emission bioprobes for live cells and in vivo imaging of animal lymphatic and other systems (Percy et al. 2009; Sojka et al. 2014). Yttrium NCs can be applied in drug release and targeted cancer cell ablation (Xu et al. 2012). The YF(3) NC-coated catheters were able to significantly reduce bacterial colonization (Lellouche et al. 2012).

Concerning the central nervous system, nanoparticles can cause both negative and positive effects on neurons (Yang et al. 2010). It is suggested that nanoparticles can have potential functional and toxicity effects on human nerve cells due to their ability to pass through biological membranes (Brooking et al. 2001). Recently, we have demonstrated that d-mannose-coated superparamagnetic maghemite (γ-Fe_2_O_3_) nanoparticles (Borisova et al. 2014) did not affect key characteristics of glutamatergic neurotransmission. In contrast, native physiologically available and synthesized in organism nanoparticles, that is, ferritin, which contains magnetic core and protein shell, affected synaptic neurotransmission (Borysov et al. 2014).

In the central nervous system of mammals, glutamate and γ-aminobutyric acid (GABA) are key excitatory and inhibitory neurotransmitters, respectively, playing primary roles in many aspects of normal brain functioning. Anomalous glutamate and GABA homeostasis contributes to neuronal dysfunction and is a characteristic feature of the pathogenesis of major neurological disorders. Exocytosis, the last step of which is fusion of synaptic vesicles containing neurotransmitter with the plasma membrane of presynaptic nerve terminals, is considered the main mechanism of neurotransmitter release. Synaptic vesicles are the acidic compartments of nerve terminals, which store the neurotransmitter and release their contents by means of exocytosis upon stimulation. Transport of glutamate, GABA, acetylcholine, monoamines and glycine, to the synaptic vesicles, is mediated by special vesicular transporters of the neurotransmitters and depends on the proton electrochemical gradient ΔμH^+^ generated by vesicular ATPase, which electrogenically pumps protons into synaptic vesicle interior. In norm, the extracellular level of GABA and glutamate between the episodes of exocytotic release is very low, thereby preventing continual activation of pre/postsynaptic receptors of these neurotransmitters (Borisova 2016). A low extracellular concentration of glutamate and GABA is maintained by specific high-affinity Na^+^-dependent transporters of the neurotransmitters, which mediate their reuptake from the synaptic cleft to the cytosol (Borisova and Borysov 2016; Borisova et al. 2016). Transporters are able to terminate synaptic neurotransmission. The transporters of glutamate and GABA belong to different families, i.e. the first ones belong to the SLC1 family, whereas the second ones (as well as carriers for the biogenic monoamines and glycine) belong to the SLC6 family. The transporters exploit Na^+^/K^+^ gradients in the plasma membrane as a driving force.

There is vast data on the optical and structural properties of NaYF_4_ nanocrystals doped with rare earth ions (Liu et al. 2013; Banski et al. 2014; Noculak et al. 2015). However, there is lack of data on their biological effects, and in particular, neuroactive effects are completely missed, despite of their great significance for theranostics and neurosurgery. The aim of the study was to assess neuromodulatory properties of synthesized hydrophilic yttrium and sodium fluoride-based NCs doped with Eu^3+^ ions. Herein, we analysed effects of NCs on the key characteristics of glutamatergic and GABA neurotransmission in presynaptic rat brain nerve terminals (synaptosomes). In this context, the following key parameters of synaptic transmission were studied: (1) uptake of glutamate and GABA via high-affinity Na^+^-dependent plasma membrane transporters using radiolabeled l-[^14^C]glutamate and [^3^H]GABA, (2) acidification of synaptic vesicles using pH-sensitive fluorescent dye acridine orange and (3) the membrane potential of the plasma membrane using potential-sensitive fluorescent dye rhodamine 6G. Previous data obtained with nerve terminals is of value for not only neurochemistry, neuromedicine and brain imaging but also can expand insight on the NCs’ ability to change the membrane potential, functional state of cellular acidic compartments, exocytosis and endocytosis of many other types of the cells.

## Materials and methods

### Materials

All synthesis and functionalization chemicals were purchased from Sigma Aldrich (USA) and used as received. Aminooxyacetic acid, EDTA, HEPES, Whatman GF/C filters and analytical grade salts were purchased from Sigma (USA). Acridine orange and rhodamine 6G were obtained from Molecular Probes (USA). Ficoll 400, l-[^14^C]glutamate, aqueous counting scintillant (ACS) and organic counting scintillant (OCS) were from Amersham (UK). [^3^H]GABA (γ-[2,3-^3^H(*N*)]-aminobutyric acid) was from Perkin Elmer, Waltham, MA (USA).

### Synthesis of NCs

The hydrophobic NCs were synthesized from chloride precursors in assistance of oleic acid and octadecene by the method described elsewhere (Ostrowski et al. 2012). In short, YCl_3_ and EuCl_3_ solved in methanol and oleic acid were added to a tri-necked flask under inert atmosphere. After a while, sodium oleate and 1-octadecane were added to the solution, and then were followed by ammonium fluoride. The synthesis solution was stirred at ~300 °C for 1 h. The final product was collected by centrifugation and washed several times with isopropanol.

### Surface functionalization of NCs

The phase transfer of NCs form hydrophobic to hydrophilic was performed by either ligand attraction or ligand protonation method for samples terminated with poly(ethylene glycol) (PEG) or hydroxyl group (OH), respectively. In brief, for the ligand attraction method, NCs were added to PEG solution and stirred for 24 h. Solvent was partially evaporated, and NCs coated with PEG were dispersed in water and sonicated. Excess water was evaporated to obtain the final solution. The ligand protonation required diluted HCl addition to the hydrophobic NCs. After 30 min, the solution was washed with water several times before final dispersion in water.

### Biological experiments

#### Isolation of nerve terminals (synaptosomes) from the rat brain

The cerebral hemispheres were rapidly removed and homogenized in ice-cold 0.32 M sucrose, 5 mM HEPES-NaOH, pH 7.4 and 0.2 mM EDTA. The synaptosomes were prepared by differential and Ficoll 400 density gradient centrifugation of the homogenate according to the method of Cotman (1974) with slight modifications (Borisova and Krisanova 2008). All manipulations were performed at +4 °C. The synaptosomal suspension was used in the experiments during 2–4 h after isolation. The standard salt solution was oxygenated and contained (in mM): NaCl 126; KCl 5; MgCl_2_ 2.0; NaH_2_PO_4_ 1.0; CaCl_2_ 2.0 and HEPES 20, pH 7.4 and d-glucose 10. Protein concentrations were measured according to Larson et al. (1986).

#### Measurements of l-[^14^C]glutamate uptake by synaptosomes

The uptake of l-[^14^C]glutamate by synaptosomes was measured as follows. The synaptosomal suspensions (125 μl; of the suspension, 0.2 mg of protein/ml) were preincubated in the standard salt solution at 37 °C for 8 min, then NCs (0.5–7.5 mg/ml) were added to the synaptosomal suspensions and incubated for 10 min. The uptake was initiated by the addition of 10 μM l-glutamate supplemented with 420 nM l-[^14^C]glutamate (0.1 μCi/ml), and incubated at 37 °C during different time intervals (1, 2 and 10 min), and then rapidly sedimented using a microcentrifuge (20 s at 10,000×*g*). The l-[^14^C]glutamate uptake was determined as a decrease in radioactivity in aliquots of the supernatants (100 μl) and an increase in radioactivity of the pellets (SDS treated) measured by liquid scintillation counting with ACS scintillation cocktail (1.5 ml) (Borisova et al. 2010).

#### Measurements of [^3^H]GABA uptake by synaptosomes

The synaptosomes were diluted in the standard salt solution containing GABA transaminase inhibitor aminooxiacetic acid at a concentration of 100 μM to minimize formation of GABA metabolites. Concentration of protein in the synaptosomal samples was 200 μg/ml. The samples were preincubated at 37 °C for 8 min, then NCs (0.5–7.5 mg/ml) were added to the synaptosomal suspension and incubated for 10 min. Uptake was initiated by the addition of GABA and [^3^H]GABA (1 μM and 50 nM–0.1 μCi/ml, respectively). The GABA uptake was terminated in different time intervals (1, 5 and 10 min) by filtering aliquots through the Whatman GF/C filters. After twice washing, with 5 ml the standard salt solution, filters were dried, then were suspended in organic counting scintillant and counted in a Delta 300 (Tracor Analytic, USA) scintillation counter (Pozdnyakova et al. 2015). Non-specific binding of [^3^H]GABA was evaluated in cooling samples sedimented immediately after the addition of radiolabeled GABA. Each measurement was performed in triplicate.

#### Assessment of the ambient level of l-[^14^C]glutamate in the preparations of synaptosomes

The synaptosomes were diluted in the standard saline solution to reach the concentration of 2 mg of protein/ml, and after preincubation at 37 °C for 10 min, they were loaded with l-[^14^C]glutamate (1 nmol/mg of protein, 238 mCi/mmol) in oxygenated standard saline solution at 37 °C for 10 min. After loading, the suspensions were washed with 10 volumes of ice-cold standard saline solution; the pellets were resuspended in the solution to a final concentration of 1 mg protein/ml and immediately used for release experiments. The synaptosomal suspensions (125 μl; 0.5 mg of protein/ml) were preincubated for 10 min, then the NCs (0.5–7.5 mg/ml) were added at 37 °C and incubated for different time intervals (0 and 6 min), and then rapidly sedimented using a microcentrifuge (20 s at 10,000×*g*). The release was measured in the aliquots of the supernatants (100 μl) and the pellets by liquid scintillation counting with scintillation cocktail ACS (1.5 ml). The results were expressed a percentage of total amount of radiolabeled neurotransmitter incorporated (Borisova 2013).

#### Assessment of the ambient level of [^3^H]GABA in the preparations of synaptosomes

The synaptosomes were diluted in a standard saline solution to 2 mg of protein/ml and after preincubation for 10 min at 37 °C were loaded with [^3^H]GABA (50 nM, 4.7 μCi/ml) in the oxygenated standard saline solution for 10 min. Aminooxyacetic acid at a concentration of 100 μM was present throughout all experiments of [^3^H]GABA loading and release. After loading, the suspensions were washed with 10 volumes of ice-cold oxygenated standard saline solution. The pellets were resuspended in the standard saline solution to obtain protein concentration of 1 mg of protein/ml. The synaptosomes (120 μl of the suspension) were preincubated for 10 min with NCs (0.5–7.5 mg/ml) at 37 °C and then rapidly sedimented using a microcentrifuge (10,000×*g*, 20 s). [^3^H]GABA radioactivity was measured in the aliquots of supernatants (90 μl) by liquid scintillation counting with scintillation cocktail ACS (1.5 ml) and expressed as percentage of a total [^3^H]GABA accumulated.

#### Measurement of synaptosomal plasma membrane potential (*E*_m_)

Membrane potential was measured using rhodamine 6G, a potentiometric fluorescent dye, at a concentration of 0.5 μM based on its binding to the plasma membrane of the nerve terminals. The suspensions of the synaptosomes (0.2 mg/ml of final protein concentration) after preincubation at 37 °C for 10 min were added to a stirred thermostatted cuvette. To assess changes in the plasma membrane potential, the ratio (*F*) as an index of membrane potential was calculated as *F* = *F*_*t*_/*F*_0_, where *F*_0_ and *F*_*t*_ are the fluorescence intensities of the dye in the absence and presence of the synaptosomes, respectively. *F*_0_ was calculated by extrapolation of exponential decay function to *t* = 0. Fluorescence measurements were carried using a spectrofluorimeter Hitachi MPF-4 at 528 (excitation) and 551 nm (emission) wavelengths (slit bands 5 nm each).

#### Measurements of synaptic vesicle acidification in the synaptosomes

Acridine orange, a pH-sensitive fluorescent dye, is known to be selectively accumulated by the acid compartments of synaptosomes (synaptic vesicles) (Borisova 2014). Therefore, it was used for monitoring synaptic vesicle acidification. Fluorescence changes were measured using a Hitachi MPF-4 spectrofluorimeter at excitation and emission wavelengths of 490 and 530 nm, respectively (slit bands 5 nm each). Reaction was started by the addition of acridine orange (final concentration—5 μM) to the synaptosomal suspension (0.2 mg/ml of the final protein concentration) preincubated in a stirred thermostatted cuvette at 30 °C for 10 min. The equilibrium level of dye fluorescence was achieved after 3 min. Fluorescence (*F*) was determined according to *F* = *F*_*t*_/*F*_0_.

### Statistical analysis

The results were expressed as mean ± SEM of *n* independent experiments. The differences between two groups were compared by two-tailed Student’s *t* test. The differences were considered significant, when *Р* ≤ 0.05.

## Results

### Structural and optical properties of NCs

**Fig. 1:**
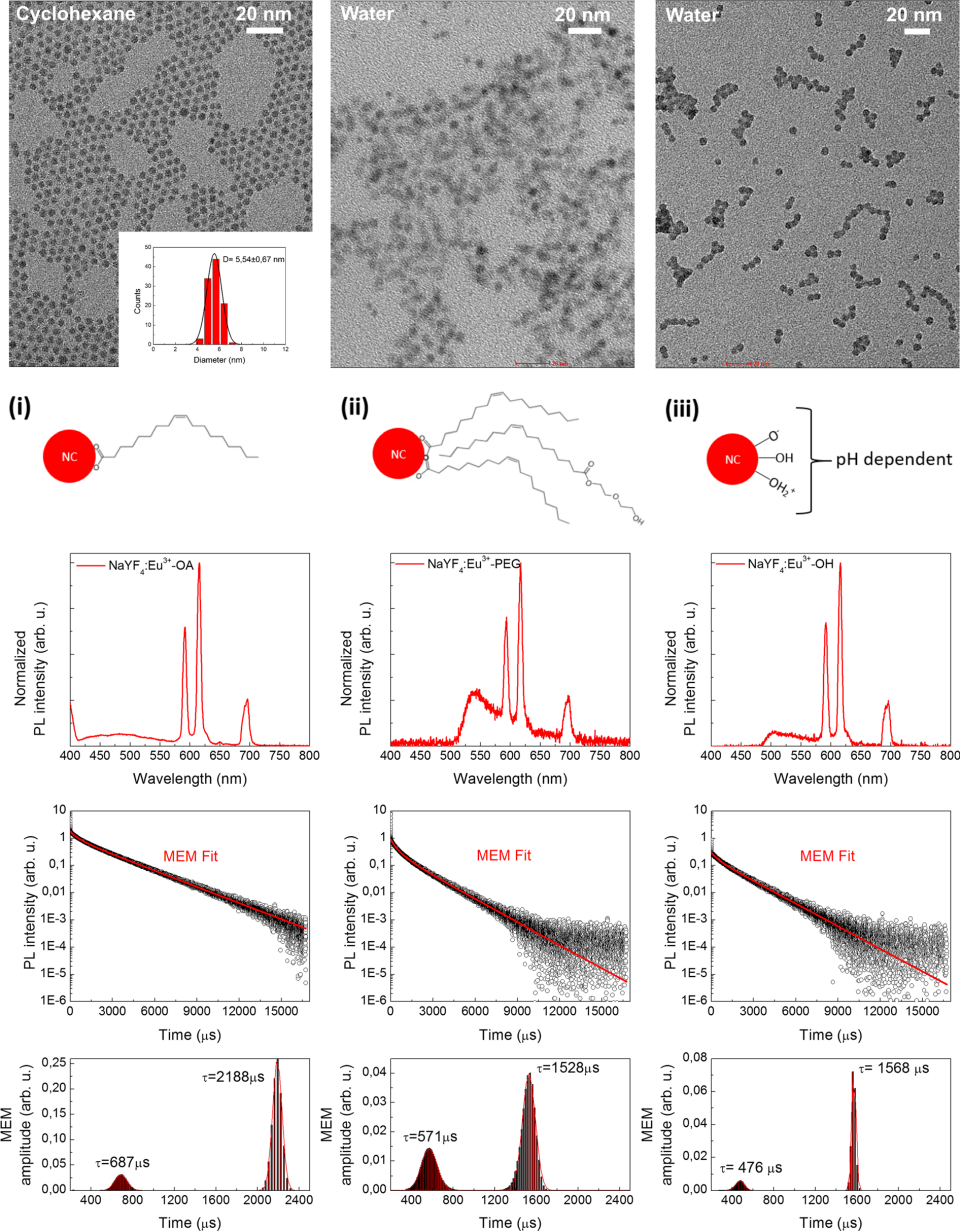
Structural, optical and temporal properties of as synthesized, functionalized with PEG-monooleate and protonated NCs (*first*, *second* and *third column*, respectively). *Top row* presents TEM pictures. *Second row* schematically depicts ligand to the NCs’ surface binding for (*i*) oleic acid, (*ii*) PEG-monooleate and (*iii*) hydroxyl group. In the *third*, *fourth* and *fifth row*, photoluminescence spectra, PL decay times and decay time distribution calculated with the Maximum Entropy Method (MEM) are presented, respectively. The emission spectra were recorded upon *λ*_exc_ = 395 nm excitation

The synthesized hydrophobic NCs were around 6 nm in diameter, which is confirmed in Fig. 1a. The photoluminescence spectra of as synthesized NCs are presented in the bottom line of Fig. 1. One can clearly see the local maxima, which correspond to the optical transitions between ^5^D_0_-^7^F_1_ (590 nm) and ^5^D_0_-^7^F_2_ (615 nm) energy levels in Eu^3+^ ions. Results for hydrophilic nanocrystals prepared according to ligand attraction and protonation methods are presented in Fig. 1, second and third columns, respectively. In the fourth row of Fig. 1, the photoluminescence decay times (*τ*_R_) measured for as synthesized as well as functionalized by poly(ethylene glycol) or protonated nanocrystals are shown. In the spectra, one can clearly observe two components of the decay times, which is confirmed by the lifetime’s distribution calculations obtained on the base of the Maximum Entropy Method (Podhorodecki et al. 2012) (fifth row of Fig. 1). The fast component is connected to the Eu ions that are at the surface of NCs and the long one to the Eu ions placed in the NCs’ core. This interpretation has been discussed in more detail in our recent paper (Sojka et al. 2016). It is important to note that both of these times are shortened after surface modification from hydrophobic to hydrophilic surface. This result can be explained based on the change of the refractive index (*n*) of the solvents. Cyclohexane, in which the samples are dispersed after synthesis, has *n* = 1.43, whereas water has *n* = 1.33. In Judd-Ofelt theory describing spectroscopic properties of lanthanides, *τ*_R_ is dependent on *n* according to the following relation (Eq. 1):

1 $$ \frac{1}{\tau_{\mathrm{R}}}\propto n{\left(\frac{n^2+2}{3n}\right)}^2={n}^{\prime } $$

For the slow *τ* component (connected with the core lanthanides), the ratio for NCs in cyclohexane and water can be estimated as follows:

2 $$ 0.94=\frac{2188}{1568}=\frac{\tau_{OA}}{\tau_{OH}}\propto \frac{n{\prime}_{OH}}{n{\prime}_{OA}}=\frac{1.19}{1.27}=1.39 $$

Similar results are obtained for the fast component of *τ*_R_ (to surface Eu connected). The numbers are not the same, since Eq. 2 is just an approximation made rather to show the trend not determine exact values. Nevertheless, one must be extremely careful when interpreting the *τ*_R_ values. It seems that it is not affected by the surface modification introduced in this work, because, when comparing the results obtained for NaYF_4_:Eu^3+^-PEG and NaYF_4_:Eu^3+^-OH, it is clear that*τ*_R_ is similar regardless of the surface modification, and therefore, it seems that the modification type is of secondary importance in this matter.

### Sonication of yttrium and sodium fluoride-based NCs doped with Eu^3+^ (NaYF_4_:Eu^3+^-PEG and NaYF_4_:Eu^3+^-OH)

Before starting the experiments, surface functionalized yttrium and sodium fluoride-based NCs doped with Eu^3+^ (NaYF_4_:Eu^3+^-PEG and NaYF_4_:Eu^3+^-OH) were subjected to preliminary treatment with ultrasound at 22 kHz for 1 min. Sonicated NaYF_4_:Eu^3+^-PEG and NaYF_4_:Eu^3+^-OH were used in the following experiments.

#### Influence of NCs on the functioning of high-affinity Na^+^-dependent neurotransmitter transporters in the plasma membrane

The experiments with NaYF_4_:Eu^3+^-PEG and NaYF_4_:Eu^3+^-OH were carried out using synaptosomes. The latest retain almost all properties of intact nerve terminals, such as an ability to maintain the membrane potential, accomplish active uptake of the neurotransmitters and their release by exocytosis. Synaptosomes are considered to be one of the best systems to study presynaptic events (Sudhof 2004).

### Influence of NCs on high-affinity transporter-mediated uptake of l-[^14^C]glutamate by synaptosomes

**Fig. 2:**
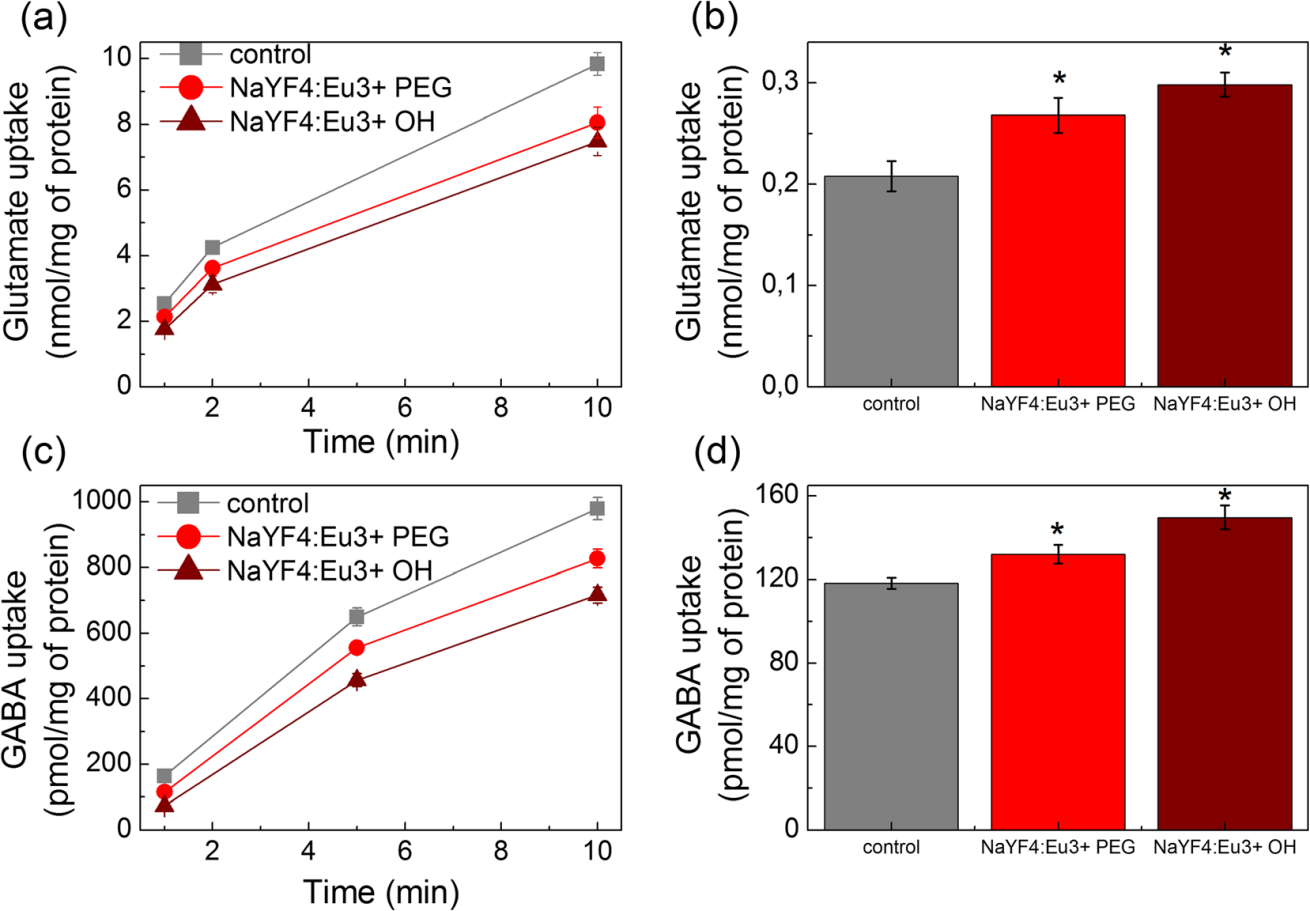
The time course of (**a**) l-[^14^C]glutamate and (**c**) [^3^H]GABA uptake by synaptosomes; extracellular level of (**b**) l-[^14^C]glutamate and (**d**) [^3^H]GABA in the synaptosomal preparations in the control, in the presence of NaYF_4_:Eu^3+^-PEG (7.5 mg/ml) and NaYF_4_:Eu^3+^-OH (3.5 mg/ml). Uptake was initiated by the addition of 10 μM l-glutamate supplemented with 420 nM l-[^14^C]glutamate (0.1 μCi/ml) or 1 μM GABA supplemented with 50 nM [^3^H]GABA (0.1 μCi/ml) to the synaptosomes (0.2 mg /ml of the final protein concentration). Data in (**a**) and (**c**) represents the mean ± SEM of six independent experiments performed in triplicate. *Single asterisk* indicates *Р* ≤ 0.05 as compared to the control

The addition of NaYF_4_:Eu^3+^-PEG and NaYF_4_:Eu^3+^-OH at a concentration range from 0.5 to 3.5 and 1.5 mg/ml, respectively, to the synaptosomes did not significantly influence the initial velocity of high-affinity sodium-dependent l-[^14^C]glutamate uptake. Whereas, further increase in the concentration of NaYF_4_:Eu^3+^-PEG and NaYF_4_:Eu^3+^-OH up to 7.5 and 3.5 mg/ml, respectively, resulted in a statistically significant decrease in above parameter. As shown in Fig. 2a, the initial velocity of l-[^14^C]glutamate uptake by synaptosomes was equal to 2.53 ± 0.02 nmol/min/mg protein in the control experiments and 2.13 ± 0.05 nmol/min/mg protein in the presence of NaYF_4_:Eu^3+^-PEG at a concentration of 7.5 mg/ml (*Р* ≤ 0.05, Student’s *t* test, *n* = 6); 1.75 ± 0.1 nmol/min/mg protein in the presence of NaYF_4_:Eu^3+^-OH at a concentration of 3.5 mg/ml (*Р* ≤ 0.05, Student’s *t* test, *n* = 6).

The accumulation of l-[^14^C]glutamate by synaptosomes for 10 min was also changed in the presence of NaYF_4_:Eu^3+^-PEG and NaYF_4_:Eu^3+^-OH (Fig. 2a) and was equal to 9.84 ± 0.35 nmol/mg protein in the control experiments and 8.06 ± 0.46 nmol/mg protein in the presence of NaYF_4_:Eu^3+^-PEG at a concentration of 7.5 mg/ml (*Р* ≤ 0.05, Student’s *t* test, *n* = 6); 7.48 ± 0.43 nmol/mg protein in the presence of NaYF_4_:Eu^3+^-OH at a concentration of 3.5 mg/ml (*Р* ≤ 0.05, Student’s *t* test, *n* = 6). Notably, before the experiments, NCs were subjected to treatment with ultrasound at 22 kHz for 1 min.

Therefore, it was shown that the NaYF_4_:Eu^3+^-PEG at a concentration range from 0.5 to 3.5 mg/ml and NaYF_4_:Eu^3+^-OH at a concentration range from 0.5 to 1.5 mg/ml did not affect l-[^14^C]glutamate uptake by synaptosomes; however, when their concentrations were increase up to 7.5 and 3.5 mg/ml, respectively, a decrease in the initial velocity of synaptosomal uptake and accumulation of l-[^14^C]glutamate was registered.

#### Influence of NCs on high-affinity transporter-mediated uptake of [^3^H]GABA by the synaptosomes

Herein, the effect of NaYF_4_:Eu^3+^-PEG and NaYF_4_:Eu^3+^-OH on the initial velocity of [^3^H]GABA uptake by the synaptosomes was assessed. NaYF_4_:Eu^3+^-PEG at a concentration range from 0.5 to 3.5 mg/ml and NaYF_4_:Eu^3+^-OH at a concentration range from 0.5 to 1.5 mg/ml did not affect significantly [^3^H]GABA uptake by synaptosomes.

As shown in Fig. 2c, NaYF_4_:Eu^3+^-PEG and NaYF_4_:Eu^3+^-OH significantly decreased the initial velocity of [^3^H]GABA uptake by the synaptosomes that consisted of 164.5 ± 6.5 pmol/min/mg protein in control and 115.5 ± 2.4 pmol/min/mg protein in the presence of NaYF_4_:Eu^3+^-PEG at a concentration of 7.5 mg/ml (*Р* ≤ 0.05, Student’s *t* test, *n* = 6); and 73.2 ± 1.7 pmol/min/mg protein in the presence of NaYF_4_:Eu^3+^-OH at a concentration of 3.5 mg/ml (*Р* ≤ 0.05, Student’s *t* test, *n* = 6).

The synaptosomal accumulation of [^3^H]GABA for 10 min was changed in the presence of NCs (Fig. 2c) and was equal to 979.6 ± 34.5 pmol/mg protein in the control experiments and 827.2 ± 27.9 pmol/mg protein in the presence of NaYF_4_:Eu^3+^-PEG at a concentration of 7.5 mg/ml (*Р* ≤ 0.05, Student’s *t* test, *n* = 6); 716.6 ± 24.3 pmol/mg protein in the presence of NaYF_4_:Eu^3+^-OH at a concentration of 3.5 mg/ml (*Р* ≤ 0.05, Student’s *t* test, *n* = 6).

Similarly with l-[^14^C]glutamate experiments, NaYF_4_:Eu^3+^-PEG at a concentration range from 0.5 to 3.5 mg/ml and NaYF_4_:Eu^3+^-OH at a concentration range from 0.5 to 1.5 mg/ml did not influence synaptosomal [^3^H]GABA uptake. At concentrations 7.5 and 3.5 mg/ml, respectively, NaYF_4_:Eu^3+^-PEG and NaYF_4_:Eu^3+^-OH became able to decrease in the initial velocity of synaptosomal uptake and accumulation of [^3^H]GABA.

There are several major factors that can influence the transporter-mediated uptake of l-[^14^C]glutamate and [^3^H]GABA by nerve terminals: (i) changes in the membrane potential as it is a driving force of neurotransmitter uptake and (ii) alterations in the proton gradient of synaptic vesicles as the latest drives accumulation of neurotransmitters by synaptic vesicles.

### Influence of NCs on the ambient level of the neurotransmitters in the preparation of synaptosomes

The maintenance of low levels of ambient glutamate and GАВА is very important for correct synaptic transmission, whereas the changes in these levels misbalance excitatory/inhibitory signals and cause neurotoxicity. Mainly, the rates of uptake determine the ambient level of glutamate and GАВА.

#### Effects of NCs on the ambient level of l-[^14^C]glutamate in the synaptosomal preparations

The effects of NCs at different concentrations on the extracellular level of l-[^14^C]glutamate in the synaptosomal suspension were assessed. It was found that NaYF_4_:Eu^3+^-PEG at a concentration range from 0.5 to 3.5 mg/ml and NaYF_4_:Eu^3+^-OH at a concentration range from 0.5 to 1.5 mg/ml did not affect significantly the extracellular level of l-[^14^C]glutamate after 10 min of incubation with synaptosomes. An increase in the concentrations of both NCs led to a raise in this parameter (Fig. 2b). The extracellular level of l-[^14^C]glutamate in synaptosomal suspension consisted of 0.208 ± 0.015 nmol/mg of protein in the control and 0.268 ± 0.017 nmol/mg of protein in the presence of NaYF_4_:Eu^3+^-PEG at a concentration of 7.5 mg/ml (*Р* ≤ 0.05, Student’s *t* test, *n* = 6) and 0.298 ± 0.012 nmol/mg of protein in the presence of NaYF_4_:Eu-^3+^OH at a concentration of 3.5 mg/ml (*Р* ≤ 0.05, Student’s *t* test, *n* = 6). Therefore, NaYF_4_:Eu^3+^-PEG and NaYF_4_:Eu^3+^-OH at a concentrations starting from 7.5 and 3.5 mg/ml, respectively, considerably increased the ambient level of l-[^14^C]glutamate in the synaptosomes.

#### Effects of NСs on the ambient level of [^3^Н]GАВА in the synaptosomal preparations

In this set of experiments, the extracellular level of [^3^H]GABA was assessed in synaptosomes in the presence of NCs at different concentrations. After 10 min of incubation of synaptosomes with NCs (Fig. 2d), NaYF_4_:Eu^3+^-PEG at a concentration range from 0.5 to 3.5 mg/ml and NaYF_4_:Eu^3+^-OH at a concentration range from 0.5 to 1.5 mg/ml did not influence significantly the extracellular level of [^3^H]GABA. As shown in Fig. 2d, the extracellular level of [^3^H]GABA in the synaptosomal suspension was equalled to 118.3 ± 2.6 pmol/mg of protein in the control and 132.1 ± 4.4 pmol/mg of protein in the presence of NaYF_4_:Eu^3+^-PEG at a concentration of 7.5 mg/ml (*Р* ≤ 0.05, Student’s *t* test, *n* = 6) and 149.6 ± 5.7 pmol/mg of protein in the presence of NaYF_4_:Eu^3+^-OH at a concentration of 3.5 mg/ml (*Р* ≤ 0.05, Student’s *t* test, *n* = 6). Therefore, а significant increase in the extracellular level of [^3^H]GABA in the synaptosomal preparations in the presence of NCs at high concentrations was found.

### The effect of NCs on acidification of synaptic vesicles in the synaptosomes

**Fig. 3:**
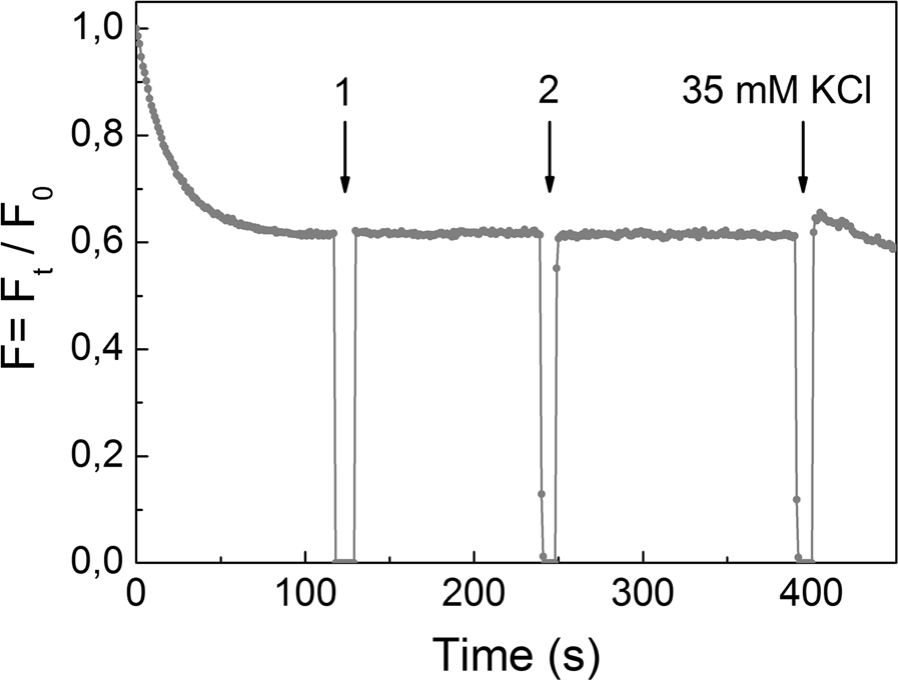
The acidification of synaptosomes in the presence of NaYF_4_:Eu^3+^-PEG. The synaptosomes were loaded with acridine orange (5 μM); when the steady level of the dye fluorescence had been reached, NaYF_4_:Eu^3+^-PEG at a concentration of 0.5 mg/ml (*arrow no. 1*) and at a concentration of 7.5 mg/ml (*arrow no. 2*) were applied to the synaptosomes. *Trace* represents three experiments performed with different preparations

Inside of nerve terminals, small amino acid neurotransmitters are accumulated in acidic cellular compartments (synaptic vesicles) by specific vesicular transporters, which use a V-type ATPase-mediated proton electrochemical gradient as а driving force. A рН-sensitive fluorescent dye acridine orange was applied in synaptic vesicle acidification measurements (Zoccarato et al. 1999). As shown in Fig. 3, the application of the dye to the synaptosomes led to partial quenching of the fluorescence signal due to dye accumulation by synaptic vesicles. After loading with acridine orange, NaYF_4_:Eu^3+^-PEG and NaYF_4_:Eu^3+^-OH were added to synaptosomes. It was demonstrated that the addition of both NaYF_4_:Eu^3+^-PEG and NaYF_4_:Eu^3+^-OH within the range of concentrations 0.5–7.5 and 0.5–3.5 mg/ml, respectively, to the synaptosomes did not influence the fluorescence intensity of acridine orange, indicating an unchanged synaptic vesicle acidification. Acidification of the synaptosomes in the presence of NaYF_4_:Eu^3+^-OH was very similar to that for NaYF_4_:Eu^3+^-PEG presented in Fig. 3, and so this data was not shown.

The addition of high-KCl to Ca^2+^-containing synaptosomal media evoked a spike of acridine orange fluorescence and then the dye reuptake representing synaptic vesicle exocytosis and endocytosis (Fig. 3). As shown in Fig. 3, the presence of NCs in the synaptosomal incubation media did not prevent KCl-induced fluorescence spike following due reuptake. Therefore, NaYF_4_:Eu^3+^-PEG and NaYF_4_:Eu^3+^-OH did not affect Ca^2+^-dependent synaptic vesicle recycling. It is suggested that during experiments, NCs can form aggregates that are too large to influence synaptic vesicle acidification. Therefore, the changes in the proton gradient of synaptic vesicles cannot be a cause of an NC-induced decrease in the initial velocity and accumulation of l-[^14^C]glutamate and [^3^H]GABA by synaptosomes.

### The plasma membrane potential of the synaptosomes in the presence of NCs

The membrane potential of the synaptosomes was measured using the cationic potentiometric dye rhodamine 6G, which is able to bind to negative charges of the membranes.

**Fig. 4:**
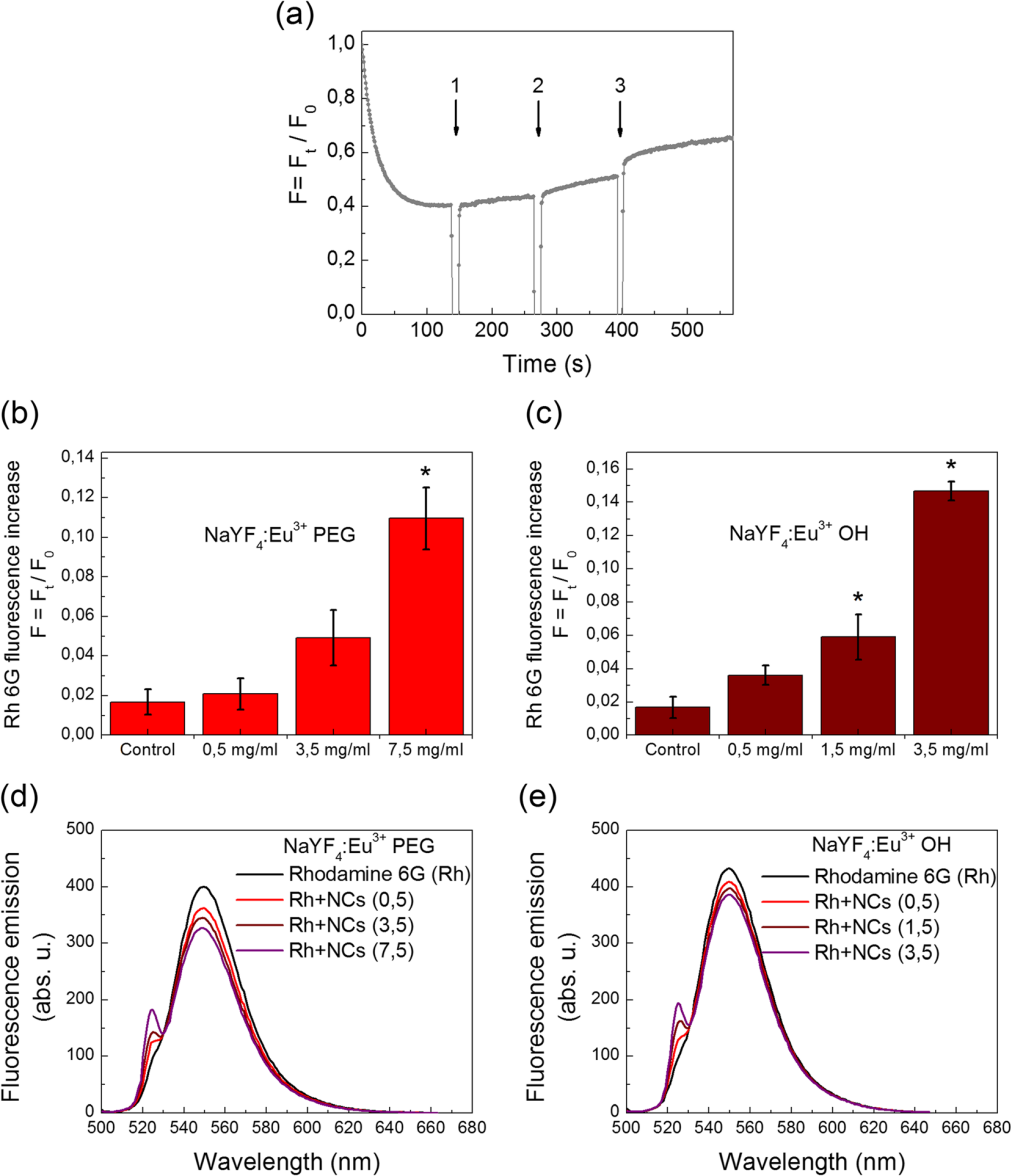
The membrane potential of the nerve terminals after the addition of NaYF_4_:Eu^3+^-PEG (**a**). The suspension of the synaptosomes was equilibrated with potential-sensitive dye rhodamine 6G (0.5 μM); when the steady level of the dye fluorescence had been reached, NaYF_4_:Eu^3+^-PEG at a concentrations of 0.5, 3.5 and 7.5 mg/ml in three additions (*arrow nos. 1*, *2 and 3*) were applied to the synaptosomes. *Trace* represents three experiments performed with different preparations. An increase in the fluorescence signal of rhodamine 6G in response to application of (**b**) NaYF_4_:Eu^3+^-PEG (0.5–7.5 mg/ml) or (**c**) NaYF_4_:Eu^3+^-OH (0.5–3.5 mg/ml), respectively, to the synaptosomes. Data is mean ± SEM. *Single asterisk* indicates *P* < 0.05 as compared to control (baseline fluorescence). Fluorescence emission spectra of rhodamine 6G (0.5 μM) in the standard salt solution before and after application of (**d**) NaYF_4_:Eu^3+^-PEG (0.5–7.5 mg/ml) or (**e**) NaYF_4_:Eu^3+^-OH (0.5–3.5 mg/ml)

As shown in Fig. 4a, the addition of the synaptosomal suspension to the medium containing rhodamine 6G was accompanied by а partial reduction in the fluorescence signal due to dye binding to the plasma membrane. *F*_st_, the membrane potential index at the steady state level, was achieved for 2 min. NaYF_4_:Eu^3+^-PEG and NaYF_4_:Eu^3+^-OH at а concentration of each NCs equal to 0.5 mg/ml did not influence the fluorescence signal of rhodamine 6G, reflecting the absence of depolarization of the synaptosomal plasma membrane (Fig. 4a–c). The dynamics of changes in the membrane potential of synaptosomes in the presence of NaYF_4_:Eu^3+^-OH was very similar to that for NaYF_4_:Eu^3+^-PEG presented in Fig. 4a, and so this data was not demonstrated. The addition of NaYF_4_:Eu^3+^-PEG at the concentrations starting from 3.5 mg/ml (Fig. 4b) and NaYF_4_:Eu^3+^-OH starting from 1.5 mg/ml (Fig. 4c) caused a dose-dependent increase in the membrane potential of the nerve terminals.

The question rose whether or not NCs influenced the fluorescence of rhodamine 6G. The emission spectrum of rhodamine 6G was not changed after application of NaYF_4_:Eu^3+^-PEG at concentrations of 0.5–7.5 mg/ml (Fig. 4d) or NaYF_4_:Eu^3+^-OH at concentrations of 0.5–3.5 mg/ml (Fig. 4e). However, at around 525 nm, a second local maximum is becoming more evident along with the increase of NC concentration and, therefore, is associated with them.

Therefore, effects of NCs on the potential of the plasma membrane can be one of the causes that led to a decrease in the initial velocity and accumulation of l-[^14^C]glutamate and [^3^H]GABA by synaptosomes shown in the previous subsections. It should be underlined that unsonicated NCs had lesser effects on the membrane potential that can be associated with their possible aggregation.

### Surface stability of the NCs

**Fig. 5:**
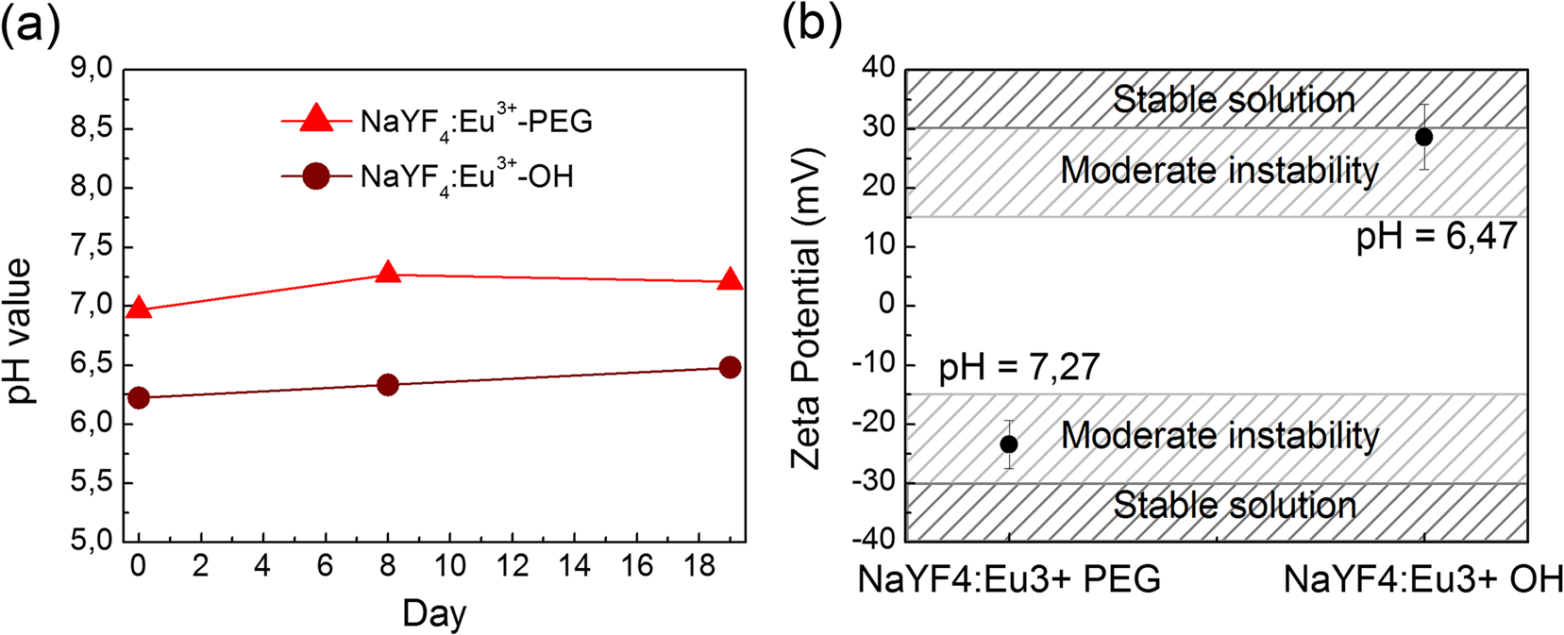
Zeta potential (**a**) and pH stability (**b**) measurements for NaYF_4_:Eu^3+^-PEG and NaYF_4_:Eu^3+^-OH NCs. The *light grey zone* is the area, in which water solutions of nanoparticles are considered not stable and tend to form aggregates

The zeta potential was measured in order to examine the NCs’ tendency to form aggregates (Fig. 5a). To determine if the NCs were stable during the experiments, their pH values (Fig. 5b) were measured over the course of 3 weeks.

## Discussion

Owing to their unique optical properties, NCs have attracted much attention in nanobiotechnology and biomedical fields, e.g. biosensing, drug delivery and in vitro and in vivo imaging (Podhorodecki et al. 2014). Advantage of NCs is based on simple and cheap fabrication, e.g. hydrolysis, solvothermal techniques, co-thermolysis or precipitation. NCs are no-blinking and no-bleaching, and their emission can be tuned spectrally from ultraviolet to infrared using both down-converting and up-converting mechanisms (Wu et al. 2009; Li and Zhang 2010; Wang et al. 2011; Teng et al. 2012; Luo et al. 2013). A possibility of combining optical, magnetic and other properties in one probe is also very perspective (Lim et al. 2012). These properties make them very promising candidates for clinical use.

In this study, we found that except fluorescent properties, yttrium and sodium fluoride-based NCs doped with Eu^3+^ (at high concentrations) possess neuroactive properties. At concentrations of NaYF_4_:Eu^3+^-PEG and NaYF_4_:Eu^3+^-OH starting from 7.5 to 3.5 mg/ml, respectively, they are able to (i) attenuate the initial velocity of uptake and accumulation of l-[^14^C]glutamate and [^3^H]GABA by nerve terminals (Fig. 2a, c) and (ii) elevate ambient l-[^14^C]glutamate and [^3^H]GABA in the preparations of nerve terminals (Fig. 2b, d). NaYF_4_:Eu^3+^-PEG and NaYF_4_:Eu^3+^-OH did not affect acidification of synaptic vesicles (Fig. 3) but caused depolarization of the plasma membrane of nerve terminals (Fig. 4a). It should be noted that NCs were preliminary treated with ultrasound to prevent their aggregation. The ability of NaYF_4_:Eu^3+^-PEG and NaYF_4_:Eu^3+^-OH at concentrations 7.5 and 3.5 mg/ml to decrease the initial rate of transporter-mediated uptake of glutamate and GABA by nerve terminals and accumulation of these neurotransmitters and significantly increase their extracellular level can led to exotoxicity. It should be noted that weak uptake can be accompanied (Borisova 2013) or not (Borisova et al. 2004; Borisova and Himmelreich 2005) by an enlargement of the extracellular level of neurotransmitters in the nerve terminals.

Recently, we performed experiments with different types of nanoparticles regarding their ability to influence key characteristics of neurotransmission using analogical methodological approach. The data obtained under similar experimental conditions allows to compare neuroactive properties of different nanoparticles. Carbon dots included in our comparative data analysis are newly discovered carbon fluorescent nanoscale particles. They are composed of a highly defected coexisting aromatic and aliphatic regions, the elementary constituents of which are graphene, graphene oxide and diamond (Gadipelli and Guo 2014). Fluorescence emission of carbon dots is typically observed in the blue and green spectrum ranges. We revealed that carbon dots synthesized from β-alanine possessed neuroactive properties at concentrations of 0.08–0.800 mg/ml (Borisova et al. 2015). The next type of nanoparticles involved in the comparative analysis is detonation nanodiamonds. They are essentially composed of carbon sp^3^ structures in the core with sp^2^ and disorder/defect carbons on the surface (Pozdnyakova et al. 2016). Nanodiamonds revealed an ability to alter key parameters of presynaptic glutamate and GABA transport at concentrations higher than 0.5 mg/ml. The third compound, neuroactive features of which plan to be compared to NCs, is ferritin. It represents a protein globule with a cavity where Fe^3+^ ions are deposited in mineral crystallites resembling ferrihydrite (Andrews et al. 1992). Ferritin core exhibits superparamagnetic properties inherent to magnetic nanoparticles (Dubiel et al. 1999), and its average diameter varies in different tissues from 3.5 to 7.5 nm (Dubiel et al. 1999; May et al. 2010). Ferritin stores cellular iron in a dynamic manner, allowing the release of the metal according to demand. We found that exogenous ferritin at a concentration of 0.08 mg/ml (iron content 0.7%) demonstrated neuroactive features (Borysov et al. 2014). The fourth type of nanoparticles included in the comparative analysis is native dust particles derived from ash deposit of fallen volcanic air (JSC-1a, and JSC, Mars-1A from ORBITEC Orbital Technologies Corporation). The average size of these particles in the suspension in standard salt solution after sonication was equal to 1110 ± 67 nm for JSC-1a and 4449 ± 1030 nm for JSC, Mars-1A, and minor fractions of nanoparticles with the size of 50–60 nm were found. Major elemental composition of JSC-1a is (in %) SiO_2_ (46.67), Al_2_O_3_ (15.79), Fe_2_O_3_ (12.5), FeO (8.17), MgO (9.39), CaO (9.9), Na_2_O (2.83), TiO_2_ (1.71), K_2_O (0.78), P_2_O_5_ (0.71) and MnO (0.19). Composition JSC is (in %) SiO_2_ (34.5), Fe_2_O_3_ (19), Al_2_O_3_ (18.5), FeO (2.5), MgO (2.5), CaO (5), Na_2_O (2), TiO_2_ (3), K_2_O (0.5), P_2_O_5_ (0.7) and MnO (0.2). Interestingly, both JSC-1a and JSC have no effects on the synaptosomal uptake and ambient level of glutamate at a concentration of 2 mg/ml. Only changes in unspecific glutamate binding to synaptosomes in the presence of JSC-1a were found. Summarizing, carbon dots (Borisova et al. 2015), detonation nanodiamonds (Pozdnyakova et al. 2016) and physiological nanocomplex ferritin exhibited neuroactivity at concentrations of 0.08, 0.5 and 0.08 mg/ml, respectively (Borysov et al. 2014). Whereas, the ability of NaYF_4_:Eu^3+^-PEG and NaYF_4_:Eu^3+^-OH to influence neurotransmitter transport was registered at concentrations starting from 7.5 to 3.5 mg/ml. Therefore, NCs start to exhibit their neuroactive effects at concentrations several times higher than those shown for above nanoparticles, and so NCs can be considered lesser neurotoxic as compared to carbon dots, detonation nanodiamonds and exogenous ferritin.

The main uncertainly of the study is the fact that both NCs can form aggregates at the time of the experiments, because their zeta potential is not optimal (meaning it is within the −30 to +30 mV limit). Although, NCs with hydroxyl groups (OH) come close to this stability range (it is within their measure error). Despite preliminary sonication of NCs performed before each experiment, it is unclear how fast their aggregation occurs in the media containing suspension of nerve terminals. It should be noted that “fresh”, newly synthesized NCs were more neuroactive (approximately by 20%) than that after several weeks of storage. The experimental data of the “Results” section represents the average meanings of the effects of NCs from different synthesis and duration of storage. What is more, NCs prepared via ligand protonation method have a pH-dependent surface (could be O^−^, OH or OH_2_^+^). It is unclear if these surfaces exchanged between each other during the experiment or if one is more reactive than the others are. As it is confirmed by the pH measurements, their surface should not change spontaneously during the experiment. However, when they are transferred to a solution with strong pH difference, aggregation or emission signal quenching might occur.

## Conclusion

In conclusion, neuroactive properties of yttrium and sodium fluoride-based NCs doped with Eu^3+^, that is NaYF_4_:Eu^3+^-PEG and NaYF_4_:Eu^3+^-OH, were registered at concentrations higher than 7.5 and 3.5 mg/ml. However, within the concentration range 0.5–3.5 and 0.5–1.5 mg/ml, NaYF_4_:Eu^3+^-PEG and NaYF_4_:Eu^3+^-OH, respectively, did not influence significantly Na^+^-dependent transporter-dependent l-[^14^C]glutamate and [^3^H]GABA uptake and the ambient level of the neurotransmitters in the nerve terminals. NaYF_4_:Eu^3+^-PEG and NaYF_4_:Eu^3+^-OH at concentrations of 7.5 and 3.5 mg/ml, respectively, did not influence acidification of synaptic vesicles but decreased the potential of the plasma membrane of nerve terminals. Comparative analysis of neuroactive features of NCs with other nanoparticles assessed with analogical methodological approach showed that NCs can be considered lesser neurotoxic as compared to carbon dots, detonation nanodiamonds and exogenous ferritin. Fluorescent and neuroactive properties of NCs can be used in neurotheranostics and neurosurgery.
